# Factors influencing participation and engagement in post-stroke cardiac rehabilitation and exercise: an exploratory qualitative study

**DOI:** 10.1097/MRR.0000000000000652

**Published:** 2025-01-24

**Authors:** Emma Martin, Trudi Cameron, Kate Radford

**Affiliations:** a School of Medicine, University of Nottingham, Nottingham; b Medical Research Council Unit for Lifelong Health and Ageing at UCL, University College London, London; c Centre for Rehabilitation and Ageing Research, School of Medicine, University of Nottingham; d NIHR Nottingham Biomedical Research Centre, Nottingham University Hospitals NHS Trust, Nottingham, UK

**Keywords:** cardiac rehabilitation, engagement, exercise, influencing, participation, post-stroke, qualitative, stroke

## Abstract

The secondary prevention benefits of cardiac rehabilitation and similar exercise classes for stroke survivors are well established, however post-stroke exercise participation remains low. This research aimed to explore the factors affecting participation and engagement in UK-based post-stroke cardiac rehabilitation and exercise, from the perspective of the service user and service provider. An exploratory study, using semi-structured interviews, was conducted (*n* = 8, service user = 4), adopting a phenomenological approach. All interviews applied a topic guide informed by the Health Belief Model and the International Classification of Functioning, Disability and Health, and were analysed using inductive thematic analysis. Post-stroke cardiac rehabilitation and exercise participation was influenced by numerous factors, encompassed into three themes: Accessibility (describing the environmental pre-class limiting factors), Programme Structure (valuing in-class supervision, socialisation and adaptations) and Patient Characteristics (encompassing the influence of the service user’s personality and experiences). Effective secondary prevention of stroke through cardiac rehabilitation and other exercise-based rehabilitation requires policy development and commissioning to ensure appropriate delivery. Further research should determine the feasibility of novel exercise class formats, in addition to larger trials investigating their clinical benefit and cost effectiveness.

## Introduction

Stroke is a leading cause of disability [1,2], inducing significant personal, functional and psychological challenges. The economic burden of stroke is also substantial, with current UK-based costs for new-onset strokes around £26 billion annually [3]. Moreover, as stroke prevalence is projected to increase by 34% by 2035 [4], and the cumulative 10-year risk of recurrence is 39% [5], this exacerbates the personal and societal stroke-related consequences. Secondary prevention is paramount to avert up to 40% of recurrent strokes [6] and diminish these negative implications.

Physical activity is one risk factor that can be targeted in stroke secondary prevention, as highlighted by international studies, including INTERSTROKE [7] and SAMMPRIS [8]. Research also highlights exercise is protective against stroke recurrence [9], where the numerous benefits culminate in a reduced 3-year risk of recurrence [10,11]. The benefits contributing to this reduced risk include aerobic capacity improvements [12–14], lean mass increases [12,13,15], cardiovascular risk factor reductions [16,17], including blood pressure reductions [18], and improved quality of life [19]. However, policy drivers, such as the National Institute for Health and Care Excellence (NICE) Stroke Rehabilitation guidelines [20] and the National Health Service Long-Term Plan [21] fail to address post-stroke secondary prevention, and only 42% of stroke survivors meet post-stroke physical activity guidelines [15]. Therefore, secondary prevention provision may be limited by this policy deficit, as well as current rehabilitation prioritising pharmacological and functional therapy [22]. Structured exercise classes, such as cardiac rehabilitation, offer an opportunity to break this trend in inactivity [15].

Cardiac rehabilitation involves personalised aerobic group exercise and education sessions [14]. These programmes are well-established in cardiovascular care [23], reducing cardiovascular morbidity and mortality [24]. However, cardiac rehabilitation also represents an effective post-stroke secondary prevention intervention, as it is noted to be safe [25,26], with significantly lower stroke reoccurrence [27] and improved post-stroke wellbeing [15]. Cardiac rehabilitation is also cost-effective, with implementation costs per quality-adjusted life year below the £20 000 NICE threshold [28]. But, despite the benefits, provision of post-stroke cardiac rehabilitation and exercise varies. In the UK, only 5% of all cardiac rehabilitation participants identify with stroke [29]. Therefore, there is a need to increase engagement and understand the factors influencing participation in post-stroke cardiac rehabilitation.

Very few studies have explored stroke survivors’ experiences of cardiac rehabilitation. From the service user perspective, social comparison to others can negatively influence the service user’s experience, whilst positive interactions with staff can promote integration [30]. However, these reports focused on the US system, limiting generalisability to the UK [30]. Moreover, from the service provider perspective, research has reported the challenges of post-stroke cognitive impairment and adaptions to each individual’s needs [31], indicating difficulties in implementation.

Therefore, whilst some research investigating the experience of stroke survivors in cardiac rehabilitation and the factors influencing post-stroke cardiac rehabilitation implementation exists, no studies have investigated the factors influencing participation and engagement in post-stroke cardiac rehabilitation and similar exercise classes, from the perspective of both service users and service providers in the UK. The aim of this study was to explore the factors affecting participation and engagement in UK-based post-stroke cardiac rehabilitation and exercise classes, from the service user’s and service provider’s perspective. This focus was crucial to understand how to maximise engagement in post-stroke cardiac rehabilitation, to influence clinical guidelines and reduce secondary stroke risk.

## Methods

### Study design

An exploratory qualitative interview study was conducted, adopting a phenomenological approach to explore the in-depth lived experiences of service users and service providers, from their perspective [32]. All elements of the design were reported according to the Consolidated Criteria for Reporting Qualitative Research guidelines [33].

### Recruitment

Service users and service providers of post-stroke cardiac rehabilitation or similar exercise classes were recruited, to explore the differing perspectives and to identify shared influential factors. Inclusion and exclusion criteria are described in Table 1.

**Table 1:** Inclusion and exclusion criteria for service users and service providers

	Service users	Service providers
Inclusion	• Aged 18 or older • Previously admitted to hospital with a stroke (ischaemic or haemorrhagic of all severities) • Participated in UK-based CR or similar exercise class post-stroke • Capacity to provide informed consent • Sufficient English proficiency to contribute to data collection	• Experience in organising and/or running UK-based post-stroke CR or similar exercise class • Capacity to provide informed consent • Sufficient English proficiency to contribute towards data collection
Exclusion	• Severe aphasia post-stroke, affecting participation in a conversation	• N/A

CR, cardiac rehabilitation.

Participants were recruited from the community, utilising multiple strategies and snowball sampling. An informal network of known academics, including academics from the social and professional networks of authors K.R. and T.C., were used to identify a wider network of experts, who were asked to approach potential participants with experience of post-stroke cardiac rehabilitation or similar exercise. Additionally, known contacts in charitable organisations were asked to advertise the research through online blogs. Recruitment also involved social media advertising, including stroke and cardiac rehabilitation-specific support groups. Through the recruitment process, participants were informed that the research was part of the first author’s dissertation research project, supervised by author K.R.

Recruitment continued until data saturation was achieved in analysis, where no new themes were evolving from the data.

### Data collection

Following informed consent, semi-structured interviews were conducted [34] by the first author, who was not previously known to the participants. This methodology allowed for an in-depth exploration of each participant’s unique post-stroke experience, facilitating more detailed data collection than could have been achieved using questionnaire-based techniques. The one-to-one environment also allowed participants to talk candidly about their experiences, facilitating discussion of more sensitive issues, not possible in a focus group setting, whilst limiting worries of judgement and communication deficits, supporting participation. Moreover, given the nature of recruitment was from a variety of sources over a period of time, it was more pragmatic to use interviews over focus groups.

To structure the interviews and ensure consistency, a topic guide was used, informed by the Health Belief Model (HBM) [35] and the International Classification of Functioning, Disability and Health (ICF) [36]. The HBM has previously been validated for predicting health behaviours, where it assumes human behaviour is driven by the individual’s health beliefs [35,37]. Consequently, this model was used to capture the extent to which post-stroke cardiac rehabilitation and exercise participation is driven by an individual’s beliefs. Moreover, the ICF considers multidimensional health elements, including biological and psychosocial factors, and was incorporated to aid classification of stroke and socio-contextual factors that influence participation and engagement [36,38]. Moreover, blending these theories for data collection enabled us to identify factors not previously addressed, allowing us to better understand how to effectively engage stroke survivors in exercise and identify real-world solutions.

The topics addressed are listed in Table 2 and these topics were consolidated in a theory-informed topic guide (see Supplementary Table 1, Supplemental digital content 1, http://links.lww.com/IJRR/A54, which presents the theory-informed topic guide utilised in the semi-structured interviews).

**Table 2:** Interview topics

Interview topics	
Referral and logistical access issues	Transportation issues
Class and activity structure	Personal factors influencing participation, for example, previous activity levels and daily life commitments
Mobility concerns and impact of any stroke-induced disability	Factors limiting access and widespread availability of post-stroke exercise classes

All interviews were conducted remotely, by the first author. The interviews were audio-recorded and transcribed verbatim, removing all personal information to ensure anonymity. Field notes were taken at the time of data collection. No repeat interviews were conducted.

### Data analysis

Thematic analysis was implemented at both a semantic and latent level, to capture both explicit meanings and deeper inferences [39]. All analysis was conducted manually and adopted an inductive approach. Analysis moved back and forth between the entire data set, the codes and the analysis produced. The findings were validated by the second author, who read and analysed all the transcripts independently, before meeting to discuss and agree the themes with the wider team. This process ensured validity, through investigator triangulation.

Data collection and analysis proceeded iteratively until data sufficiency was achieved. Furthermore, all authors were independent of the cardiac rehabilitation and exercise classes implemented and had no pre-conceived ideas influencing analysis.

### Ethics

This study was approved by the University of Nottingham Faculty of Medicine and Health Sciences Research Ethics Committee (ID: FMHS 195-0221) on 30 March 2021.

## Results

### Sample

Four service users and four service providers of post-stroke cardiac rehabilitation or similar exercise classes were recruited. Service providers included stroke-specific fitness instructors, physiotherapists and researchers. Interviews lasted a mean 51 min (range = 33–65 min) and were conducted between May and July 2021. No participants withdrew and all data was utilised in the analysis. Participant demographics are outlined in Table 3.

**Table 3:** Participant characteristics (numerical data, mean ± SD)

	Service users (*n* = 4)	Service providers (*n* = 4)
Sex	Male = 2	Male = 1
Age (years)	56.75 ± 7.09	53.25 ± 5.91
Time since stroke (years)	9.00 ± 4.69	N/A
Clinical role	N/A	Fitness instructor, *n* = 1 Physiotherapist/researcher, *n* = 2 Clinical lead, *n* = 1
Time in role (years)	N/A	6.50 ± 4.36

### Themes

Three main themes and 10 sub-themes were identified, presenting factors that act as enablers and barriers to post-stroke cardiac rehabilitation and exercise. The main themes were: Accessibility, Programme Structure and Patient Characteristics.

The themes are defined in Table 4.

**Table 4:** Final themes, with definitions, sub-themes and examples of sub-theme topics

Theme	Definition	Sub-themes	Examples of included topics
Accessibility	Pre-class factors regarding getting to the scheduled CR and exercise classes and discussion of how this affected participation	Transport	Loss of independence Reliance on others and public transport Geographical distance to classes Financial costs of travel
		Fatigue	ADL-related fatigue Managing fatigue Scheduling in exercise
		Alternative class timings and formats	Difficulty balancing classes with other daily commitments Limited alternative class times and formats Challenges of online class formats
		Venue suitability	Affordable parking Importance of an accessible venue
Programme structure	In-class factors, that supported or limited participation, both in the exercise class environment and at home following programme completion	Staffing	Supportive staff Patient safety reassurance from staff presence Lack of stroke-specific knowledge
		Benefits of class socialisation	Opportunity to meet other stroke survivors Socialisation aids adherence Camaraderie in a group setting
		Tailored exercise	Stroke-adapted exercise ensured inclusion compared to a standard exercise class Difficulties when adapting to each patient’s unique deficit
		Adaptability of exercises	Adapting exercises to the home environment Adapting exercises supported long-term participation benefits
Patient characteristics	Influence of each SU’s personality and experiences on their exercise engagement and participation	Pre-stroke exercise experience and understanding	Positive and negative influence of pre-stroke exercise experience Opportunity to use exercise classes to add to limited post-stroke rehabilitation
		Personality traits and priorities	Influence of each individual’s character Feelings of embarrassment post-stroke, related to stroke deficits Prioritising exercise in day-to-day life

ADL, activities of daily living; CR, cardiac rehabilitation; SU, service user.

All the sub-themes map onto the theoretical frameworks (HBM and ICF), demonstrated in Table 5.

**Table 5:** Mapping between sub-themes and theoretical models

Themes	ICF			HBM		
	Personal	Environmental	Participation	Perceived benefit	Perceived barrier	Cues to action
Accessibility						
Transport		**●**			**●**	
Fatigue					**●**	
Alternative class timings and formats	**●**	**●**				
Venue suitability		**●**				
Programme structure						
Staffing		**●**				
Benefits of class socialisation				**●**		
Tailored exercise		**●**	**●**			
Adaptability of exercise		**●**				
Patient characteristics						
Pre-stroke exercise experience and understanding	**●**			**●**		**●**
Personality traits and priorities	**●**					

HBM, Health Belief Model; ICF, International Classification of Functioning, Disability and Health.

### Accessibility

This theme included issues regarding getting to the classes, and how this created a barrier to post-stroke cardiac rehabilitation and exercise participation.

#### Transport

Service users described a loss of independence and relied on others for activities of daily living, including travel, creating a perceived barrier to participation.

‘A few participants… couldn’t drive themselves, so they had… family members bring them’. (SP3)

Environmental factors, such as the distance and travel costs, also limited attendance. Moreover, as service users were typically out-of-work, this financial pressure was emphasised.

‘People have to travel quite far in some cases’. (SU1)

‘People post-stroke, they’re not working, they haven’t got the financial means. Unless they’ve got a partner who’s working… then it’s really difficult’. (SP2)

#### Fatigue

Fatigue was another perceived barrier, often leaving service users too tired to engage with the classes.

‘If I was working full time, and then… come home to do exercise, I just wouldn’t have the energy’. (SU3)

However, through scheduling, some service users were able to manage their fatigue and maximise their engagement.

‘Because of the fatigue, you have to pace yourself more’. (SU1)

#### Alternative class timings and formats

Participants reported that classes were typically in the middle of the day and described the difficulties in balancing this with other commitments.

‘I think for her it was… being able to commit, because she… was also trying to manage her children’. (SP3)

Online resources were also limited. There was dissonance between service users, who felt online classes would lack camaraderie, and service providers, who believed lecture-style education could run online.

‘That group… sort of makes it work… You can’t do it online’. (SU1)

‘Education sessions… would be a good opportunity (to be online)’ (SP3)

#### Venue suitability

Finding a suitable venue was important to optimise participation. Ease and affordability of parking and easy building access facilitated engagement.

‘I feel for patients having to pay for parking… It’s things like that we need to look at’ (SP4)

### Programme structure

This theme encompasses in-class factors, both in the session and following programme completion.

#### Staffing

Participants were positive about the verbal and physical support from staff. Moreover, where participants felt fearful, due to concerns of injury or stroke recurrence, supervision created a safe environment and eased concerns.

‘Knowing that they had the heartrate monitor on… and qualified people observing them… there was a definite reassurance there’. (SP2)

However, as no specific guidelines for post-stroke cardiac rehabilitation exist in the UK, service providers described a lack of stroke-specific training, resulting in limited staff knowledge.

‘If they’ve got questions related to other issues like sensory deficit, ataxia… they (staff) wouldn’t have been able to answer specific stroke-related questions’. (SP2)

#### Benefits of class socialisation

Socialisation opportunities were reported positively by both groups, as a perceived benefit of participation. Participants likened the class to a ‘day-out’, as well an opportunity to build confidence, maximising the benefits.

‘It was literally getting out of the house, and the social aspect’. (SU2).

‘There was a camaraderie… they were all working hard to try to be healthy’. (SP3)

#### Tailored exercise

Participants described how exercises could be adapted, for example, exercising unilaterally for one-sided weakness. This environmental adaptation supported participation.

‘When I couldn’t do something, she would say “do it like that, do it like this”’. (SU4)

Although service users benefited from these adaptations, the complexities of personalisation for each participant were discussed, due to the uniqueness of each individual’s disability.

‘Everyone’s mobility issue is different. So you can’t just have one generic class’. (SU3)

#### Adaptability of exercises

Both service users and service providers described how exercises could be adapted to different environments. They described how this aided long-term adherence upon programme completion.

‘You could easily adapt it… milk bottle for weights, if you didn’t have the machines’. (SU2).

### Patient characteristics

This theme describes how each service user’s personality and experiences influenced their engagement.

#### Pre-stroke exercise experience and understanding

Service users with pre-stroke activity experience were more positive about post-stroke exercise, often with a greater understanding of the benefits of exercise and facilitating a cue to action to motivate participation.

‘I always did something… I’m that sort of mindset… I expect to continue to do that’. (SU3)

‘Yes I… wanted to get my activity back. I wanted the enjoyment’. (SU4)

#### Personality traits and priorities

The service user’s individual character also influenced exercise engagement, with self-belief often increasing activity. However, some service users reported embarrassment associated with their stroke-induced disability, hindering participation.

‘Quite a few say, “Well, this is my life, that’s it… I’m in a wheelchair” and I didn’t really accept that’. (SU1)

Nevertheless, engagement was dependent on individual priorities, with one service user acknowledging that prioritising exercise is a decision that everyone must consider, regardless of disability.

‘Because everyone has so many commitments… If you really want to do it, there’s a way to do it’. (SU3)

## Discussion

This study explored the factors affecting participation and engagement in UK-based post-stroke cardiac rehabilitation and exercise, from the perspective of both service users and service providers. The purpose was to understand how to maximise engagement in post-stroke cardiac rehabilitation, to influence clinical guidelines and reduce secondary stroke risk. Overall, three themes evolved, categorised into pre-class factors (Accessibility), in-class factors (Programme Structure) and factors regarding the service user’s attributes (Patient Characteristics).

### Accessibility

Transport issues were reported as an ‘Accessibility’ barrier. Environmental factors influence behaviour [36], which is consistent with our finding that travelling extensive distances impacted participation. Perceived barriers also deter participation [35], as our analysis suggested being travel-dependent on others limited engagement.

Research investigating factors influencing post-stroke physical activity found 29.9% [40] of stroke survivors believed lacking transport was a participation barrier [40]. Additionally, 53% of cardiac rehabilitation coordinators (*n* = 101) also believed transportation issues limited participation [41]. This is consistent with our findings that service users struggled to source suitable transport, travel the extensive distances to classes and fund this travel. As a result, commissioning bodies should advocate for cardiac rehabilitation and similar exercise classes, to ensure widespread implementation and to minimise these travelling distances. Furthermore, provision of transport through localised patient services could also aid engagement.

Home-based exercise programmes may help to overcome these issues [42], and this has been favoured in recent years with a 60% increase in remote cardiac rehabilitation following the COVID-19 pandemic [43]. However, our analysis suggests home-based programmes lacked popularity. Many believed a remote intervention limited group camaraderie, highlighting a personal and environmental factor limiting engagement [36]. This is in agreement with evidence from a home-based cardiac rehabilitation programme, where service users described the challenges of establishing rapport with staff [44]. Moreover, the use of technology in home-based cardiac rehabilitation may also present a barrier, with service users reporting a lack of adequate internet connection [18] and confidence using technology [44]. Furthermore, high-risk patients may be unable to access home-based cardiac rehabilitation, emphasising a lack of accessibility with remote delivery [44].

Therefore, whilst opportunities for an online or blended intervention seem promising, policy should promote interventions that can be tailored to individual needs, to optimise individual rehabilitation potential.

### Programme structure

In-class factors influencing exercise engagement were summarised into the ‘Programme Structure’ theme. Theory suggests the perceived benefits of an action can contribute to engagement [35] and our finding, that meeting similar people fostered motivation, aligns with this theory [35] and with previous studies [45,46].

Post-stroke group exercises classes are understood to enhance service user’s confidence and motivation, and seeing other achieve their goals increased engagement [46]. Further research also highlighted the importance of social support and developing relationships with other like-minded service users, to create a sense of belonging, reinforcing the importance of socialisation to increase engagement [47]. Therefore, policies should emphasise socialisation in any adopted post-stroke exercise class format.

The literature, similar to our findings, also highlights the instrumental role of service providers to facilitate exercise and empower service users, by demonstrating technique and creating a safe environment [47,48]. Moreover, 78.3% of stroke survivors in one study identified supervision as a facilitator for post-stroke exercise participation [40]. However, research also acknowledges a lack of stroke-specific staff knowledge [41,49], highlighting an environmental factor impeding participation [36]. This is consistent with discussion of professionals’ self-efficacy, where 76% had safety concerns for stroke survivors in cardiac rehabilitation [41] and many had a fear of liability [49].

Therefore, implementation of stroke-specific training could upskill staff, and as limited stroke-specific or behavioural change training currently exists, this should be incorporated into the Stroke-Specific Education Framework [49–51]. Upskilling cardiac rehabilitation staff would maximise engagement, improve delivery and increase class availability, overcoming the previous accessibility issues.

### Patient characteristics

Our analysis revealed post-stroke exercise engagement was influenced by a person’s ‘Patient Characteristics’. These personal factors influence participation [36], whilst the HBM suggests cues to action can provide motivation to provoke behaviour change [35].

Research suggests pre-stroke inactivity was the largest predictor of inactivity 1-year post-stroke, emphasising the long-term influence of inactivity on exercise engagement [52,53]. This supports our analysis, which observed those with active lifestyles pre-stroke had a greater understanding of the benefits of exercise, motivating engagement. Furthermore, studies suggest increased post-stroke exercise engagement is due to an association between pre-stroke activity and improved post-stroke functionality [54,55]. Therefore, it is possible pre-stroke exercise not only aids motivation, as in the present study, but improves functionality which supports participation. Interestingly, recent evidence also noted stroke survivors who undertook post-stroke physical activity remained more physically active 12 months later, demonstrating the ability of these classes to induce behaviour change [18,35].

Nevertheless, not all service users were positive about their rehabilitation, as some felt their disability-related embarrassment limited their engagement. This is in agreement with research noting a low self-efficacy for exercise, where service users lacked confidence in their exercise ability [56]. It is possible this uncertainty amongst service users is heightened by a lack of stroke-specific staff knowledge, highlighting a need to reinforce staff education in policy initiatives, to maximise the post-stroke secondary prevention benefits.

### Strengths and limitations

Due to the recruitment strategy, participants were largely recruited from research-based cardiac rehabilitation, indicating research is needed to understand how community-based provision can be improved. Recruitment challenges during the COVID-19 pandemic also resulted in a small sample, limiting generalisability to the wider population and potentially restricting the range of themes generated. Additionally, whilst the analysis was validated by investigator triangulation, the triangulation of multiple data collection methods could be used to strengthen and enhance the credibility of future research. However, the unique sample heterogeneity of service users and service providers widened the representativity, and the interview process was strengthened by a theory-informed topic guide, highlighting the dependability of these findings.

### Conclusion

In conclusion, few stroke survivors participate in cardiac rehabilitation or similar exercise classes and numerous factors influence post-stroke engagement. These factors include geographical limitations, accessible classes and limited stroke-specific knowledge amongst service providers. To overcome these barriers, post-stroke secondary prevention requires investment, to support an effective service delivery. Moreover, service provider upskilling should be considered, to induce exercise-related behaviour change and support. Future research should include feasibility trials testing class formats, and larger trials must test the clinical benefit and cost effectiveness of these novel services.

## Acknowledgements

The authors are grateful to all the persons who supported recruitment and to all the participants who contributed to our research findings.

### Conflicts of interest

There are no conflicts of interest.

## Supplementary Material
