# Supplementary material for: Factors influencing participation and engagement in post-stroke cardiac rehabilitation and exercise: an exploratory qualitative study
Source: Int J Rehabil Res. 2025 Jan 24;48(1):55–62. doi: 10.1097/MRR.0000000000000652 (PMC11792989; doi:10.1097/MRR.0000000000000652)
Supplement: Supplementary file 1 [file ijrr-48-55-s001.pdf]

Supplemental Digital Content 1.docx

Supplemental Digital Content 1A – Theory-Informed Topic Guide (f*or Service Users)*

Cardiac Rehabilitation Post-Stroke – Interview Topic Guide (for Service Users)

I am a Master's student working on this University of Nottingham study, which aims to explore factors influencing participation in cardiac rehabilitation/similar exercise classes in stroke survivors. You have kindly agreed to take part in our interview process and we want ask you some questions regarding your access to and experiences in cardiac rehabilitation/exercise post-stroke. We value your answers and hope, with your insight, we can improve the experience and adherence of those undertaking cardiac rehabilitation/exercise as part of their post-stroke rehabilitation in the future. Your name will not be published, and any names, locations or other identifiable information will be anonymised when transcribed. Your participation in this interview is voluntary and there are no negative implications for you.

Please can you confirm that you have read the participant information sheet and have given consent to participate?

Do you have any questions you would like to ask?

Are you still happy to go ahead with the interview and answer some questions?

QUESTIONS	PROMPTS	LINKS TO
Can you tell me about your stroke?	Do you know what type of stroke you had?<br>When did you have the stroke?<br>How did your stroke affect you? (both physically and psychologically?)<br>Has your stroke left you with any issues or functional limitations? For example, in your ability to walk, communicate, or issues with your balance or coordination?<br>Did you attend cardiac rehabilitation classes specifically? Or did you attend another type of structured exercise class?	Personal factors (ICF)
Can you tell me about how you accessed cardiac	How did you come to take part in cardiac rehabilitation/similar exercise class?<br>Were you referred?	Environmental factors (ICF)

rehabilitation/ similar exercise class?	Did you source the programme independently? If so, how did you find out about it?<br>Did you experience any problems in accessing the programme?<br>How did you feel when you accessed the programme?	 <br>Personal factors (ICF)
Can you talk me through your experience of the cardiac rehabilitation classes/similar exercise classes?	Can you describe the programme to me?<br>What was it like? How was it structured – education, exercise, relaxation?<br>Did the programme involve both exercise training and education?<br>Who delivered the training?<br>Where was it based? Were you comfortable with this environment?<br>How many people were present in the classes? Were you comfortable with this number or did you find it overwhelming?<br>Were you able to physically participate in all aspects of the programme?<br>Were all aspects suitably adapted to the needs of stroke survivors?<br>Was the education and training help useful?<br>Did you feel supported in the sessions?<br>Did you attend all the sessions?<br>Was there anything that negatively impacted your experience and stopped you from attending the classes?<br>What encouraged or discouraged you to participate?<br>If you didn't manage to complete the programme, why not?	Environmental factors (ICF)<br><br>Biological factors (ICF)<br><br>Personal factors (ICF)<br><br>Perceived benefits/barriers (HBM)
Can you talk me through any mobility issues you faced when accessing and participating in classes?	Did you feel any stroke-induced disability influenced your training and participation? Were exercises adapted?<br>How did you travel to the classes? Was this transport accessible?<br>If you had to finance this transport, is this something that limited your participation (due to financial costs)?<br>Did these issues affect your access to the classes?<br>Were you able to use all the equipment and exercise classes?<br>Were all aspects suitably adapted to your mobility needs?	Biological factors (ICF)<br><br>Environmental factors (ICF)<br><br>Personal factors (ICF)
How did your previous activity and exercise experience influence your participation?	Would you describe yourself as physically active before your stroke?<br>If you were active before, do you believe this supported your participation in these classes? How? Why?	Personal factors (ICF)

	Similarly, if you would describe yourself as inactive before, do you think this negatively impacted your participation? How? Why?<br>Did you see exercise as important in a healthy lifestyle before your stroke? If not, has this changed now?<br>Did you want to increase your activity? Did you believe you would be able to?	Perceived severity/benefits (HBM)<br>Perceived efficacy (HBM)
Tell me about your experience of fitting these exercise classes around your daily life activities.	How often did the classes run?<br>How long were each of the classes?<br>Did the classes run at the same time each week? Were there alternative times if you couldn't attend certain classes?<br>Were the classes organised at a time that worked around your other commitments (e.g. work or childcare etc.)?<br>Did the programme offer online resources as an alternative to in person classes, in the situation where timings didn't align? Would this have been helpful?<br>Did the COVID-19 pandemic impact your ability to attend the classes?	Environmental factors (ICF)
To what extent do you feel participation in these classes has changed your activity levels and healthy lifestyle behaviour post-stroke?	Did you see your stroke as a severe condition?<br>Did you want to change your exercise behaviours?<br>Do you now exercise more regularly? Do you believe this is as a result of your experiences in cardiac rehabilitation?<br>Do you know feel confident when exercising independently?<br>Do you believe the cardiac rehabilitation programme was effective for you?	Perceived severity (HBM)<br><br>Perceived efficacy (HBM)<br><br>Personal factors (HBM)

The interview is now complete. Thank you for taking part. Do you have any further questions?

If you feel you require further support to discuss the issues raised or to discuss any other issues regarding your post-stroke rehabilitation, we advise you contact your GP, speak to friends and family, or contact support associations, such as the Stroke Association.

Do you know anyone who has also suffered a stroke and experienced cardiac rehabilitation/similar exercise class? If so, would you be able to ask them to consider taking part in my research?

21 Thank you again for taking part in our research – your participation is greatly appreciated.

Supplemental Digital Content 1B – Theory-Informed Topic Guide (f*or Service Providers)*

<u>Cardiac Rehabilitation Post-Stroke – Interview Topic Guide (for Service Providers)</u>

I am a Master's student working on this University of Nottingham study, which aims to explore factors influencing stroke survivors' participation in cardiac rehabilitation and exercise. We understand you have kindly agreed to take part in our interview process and we want ask your opinions on the factors influencing stroke survivors' access to and participation in these classes. We value your answers and hope, with your insight, we can improve the experience and adherence of those undertaking cardiac rehabilitation as part of their post-stroke rehabilitation in the future. Your name will not be published, and any names, locations or other identifiable information will be anonymised when transcribed. Your participation in this interview is voluntary and there are no negative implications for you.

Please can you confirm that you have read the participant information sheet and given consent to participate?

Do you have any questions you would like to ask?

Are you still happy to go ahead with the interview and answer some questions?

QUESTIONS	PROMPTS	LINKS TO
Can you tell me about how stroke survivors accessed your cardiac rehabilitation programmes/similar exercise class?	How do stroke survivors come to take part in cardiac rehabilitation/exercise?<br>Are stroke survivors referred to you? How?<br>Can stroke survivors source the programme independently? If so, how is the programme advertised? How do stroke survivors find out about it and access it?<br>Were there any problems in stroke survivors accessing the programme?<br>What sort of issues do you think stroke survivors experience when accessing the programme?	Environmental factors (ICF)<br><br>Personal factors (ICF) / Perceived barriers (HBM)
Can you tell me more about the cardiac rehabilitation classes/ similar exercise classes that you lead?	Can you describe the programme to me?<br>What was it like? How was it structured?<br>Who delivered the training?<br>Did the programme involve both exercise training and education? If so, what topics did you cover?	Environmental factors (ICF)

	How many people were present in the classes? Was this easy to manage or should the classes be bigger/smaller?<br>Were stroke survivors able to physically participate in all aspects of the programme?<br>Did you adapt exercises to suit the needs of stroke survivors?<br>Was the education and training help well received?<br>Did you feel able to offer direct support to the stroke survivors participating?<br>Was there anything that negatively impacted participation and may have stopped a stroke survivor from attending the classes?<br>From your perspective, what encouraged or discouraged you to participate?<br>From your perspective, why do some stroke survivors not attend or complete the programme? What factors influence their participation?	Biological factors (ICF)<br><br>Personal factors (ICF)<br><br>Perceived barriers (HBM)
Can you talk me through how you cope with stroke survivors with mobility issues in the classes?	To what extent do you feel the stroke-induced disability influenced the stroke survivor's training and participation?<br>What mobility barriers do you feel there are, that affect the stroke survivor's participation in the class?<br>Were you able to accommodate any mobility issues?<br>Did you adapt the exercises to suit the participant? Are these adaptations personalised to the individual?<br>Did participants struggle to 'get to' the classes, in terms of transport? Was transport provided? Who funds the transport? Was this transport accessible?<br>If not, how did participants travel to the classes?<br>If participants had to fund their own transport, do you feel this may have limited their participation (due to financial costs) and limits who gets to take part?<br>If there any equipment you used that not everybody could access? Why is that? Or was everything physically accessible?	Biological factors (ICF)<br><br>Perceived barriers (HBM)<br><br>Environmental factors (ICF)<br><br>Perceived barriers (HBM)<br><br>Personal factors (ICF)
To what extent do you believe an individual's previous	Would you say people who were physically fit before the stroke are more likely to attend and complete the programme? Why is this?	Personal factors (ICF)

activity levels (pre-stroke) can influence or affect their participation in the classes?	Do you believe being physically active before the stroke supported stroke survivor participation in these classes? How? Why?<br>If the stroke survivor was inactive before, do you think this negatively impacted their participation? How? Why?<br>Did stroke survivors want to increase their activity? Did they believe they were able to?	Cues to action (HBM)<br><br>Perceived severity/benefits (HBM)<br>Perceived efficacy (HBM)
To what extent do you believe an individual's daily life activities affected their participation in the classes? For example, with childcare and work?	How often did the classes run?<br>How long were each of the classes?<br>Did the classes run at the same time each week? Were there alternative times if the stroke survivor couldn't attend certain classes?<br>Were the classes organised at a time that worked around people's day-to-day commitments (e.g. work or childcare etc.)?<br>Did the programme offer online resources as an alternative to in person classes, in the situation where timings didn't align? Would this be possible and helpful?<br>How has the COVID-19 pandemic impact your ability to run these classes? Is it something you can do online/remotely? Do you think this has affected the stroke survivor's participation in exercise?<br>Are there any other factors that you believe influenced who was able to access the classes?	Environmental factors (ICF)<br><br>Perceived barriers (HBM)
What (other) challenges do you perceive in delivering cardiac rehabilitation and exercise classes to stroke survivors?	Did you think stroke is too severe a condition to generate improvements in cardiovascular health?<br>Do you think stroke survivors want to change their exercise behaviours? Why?<br>From your perspective, what barriers do stroke survivors face when accessing the classes? And when taking part in the classes?<br>Do you know if the funding limits the availability of these classes?<br>Do you believe stroke survivors exercise more regularly following cardiac rehabilitation? Do you feel this is as a result of the classes? Why?<br>To what extent do you believe the classes affect the stroke survivor's confidence when exercising independently?<br>Do you believe cardiac rehabilitation programmes are effective?	Perceived severity (HBM)<br><br>Perceived barriers (HBM)<br><br>Perceived efficacy (HBM)

		Personal factors (ICF)
From your perspective, why aren't these cardiac rehabilitation classes more widely available to stroke survivors?	From your perspective, why aren't these cardiac rehabilitation classes more widely available?<br>Are you aware of any clinical guidelines, national policy or strategic plans promoting the use of cardiac rehabilitation and exercise in stroke survivors?	

The interview is now complete. Thank you for taking part. Do you have any further questions?

Do you know anyone else who would be interested in taking part in my research? Either a stroke survivor who has participated in cardiac rehabilitation/exercise post-stroke or a service provider?

Thank you again for taking part in our research – your participation is greatly appreciated.

39
